# Integrative analysis of single-cell transcriptomic and multilayer signaling networks in glioma reveal tumor progression stage

**DOI:** 10.3389/fgene.2024.1446903

**Published:** 2024-11-13

**Authors:** Fereshteh Fallah Atanaki, Leila Mirsadeghi, Mohsen Riahi Manesh, Kaveh Kavousi

**Affiliations:** ^1^ Laboratory of Complex Biological Systems and Bioinformatics (CBB), Department of Bioinformatics, Institute of Biochemistry and Biophysics (IBB ), University of Tehran, Tehran, Iran; ^2^ School of Engineering, Campbell University, Buies Creek, NC, United States

**Keywords:** scRNA-seq, tumor microenvironment, glioma progression, inter-intra signaling network, machine learning

## Abstract

**Introduction:**

Tumor microenvironments (TMEs) encompass complex ecosystems of cancer cells, infiltrating immune cells, and diverse cell types. Intercellular and intracellular signals within the TME significantly influence cancer progression and therapeutic outcomes. Although computational tools are available to study TME interactions, explicitly modeling tumor progression across different cancer types remains a challenge.

**Methods:**

This study introduces a comprehensive framework utilizing single-cell RNA sequencing (scRNA-seq) data within a multilayer network model, designed to investigate molecular changes across glioma progression stages. The heterogeneous, multilayered network model replicates the hierarchical structure of biological systems, from genetic building blocks to cellular functions and phenotypic manifestations.

**Results:**

Applying this framework to glioma scRNA-seq data allowed complex network analysis of different cancer stages, revealing significant ligand‒receptor interactions and key ligand‒receptor-transcription factor (TF) axes, along with their associated biological pathways. Differential network analysis between grade III and grade IV glioma highlighted the most critical nodes and edges involved in interaction rewiring. Pathway enrichment analysis identified four essential genes—*PDGFA* (ligand), *PDGFRA* (receptor), *CREB1* (TF), and *PLAT* (target gene)—involved in the Receptor Tyrosine Kinases (RTK) signaling pathway, which plays a pivotal role in glioma progression from grade III to grade IV.

**Discussion:**

These genes emerged as significant features for machine learning in predicting glioma progression stages, achieving 87% accuracy and 93% AUC in a 3-year survival prediction through Kaplan-Meier analysis. This framework provides deeper insights into the cellular machinery of glioma, revealing key molecular relationships that may inform prognosis and therapeutic strategies.

## 1 Introduction

Glioma is the most common type of central nervous system tumor and is derived from non-neuronal glial cells. According to The World Health Organization (WHO) gliomas are broadly categorized into three types: IDH wild-type (WT), IDH mutant (MUT) with 1p/19q co-deletion, and IDH mutant (MUT) without 1p/19q co-deletion (Byun and Park, 2022). The higher the WHO grade, the worse the prognosis, and the more aggressive the tumor. Glioblastoma is a grade IV glioma that arises from astrocytes. It is the most aggressive and lethal type of primary brain tumor. The overall survival rate of glioblastoma multiform (GMB) patients is still only 12–18 months, even with different types of medical treatments (Minniti et al., 2008). Given the poor prognosis of glioblastoma patients, it is crucial to deepen our understanding of this cancer by examining the complex interactions within the tumor microenvironment. This knowledge is essential for developing more effective treatments.

Tumor microenvironments (TME) is a complex system of multiple cell types, including fibroblasts, blood vessels, immune cells, and extracellular matrix, as well as signal molecules and mediators. These various cell types interact with one another, and their combined effects define the course of the disease and the effectiveness of the treatment (Wang et al., 2024; Dominiak et al., 2020). In fact, tumor cells recruit and rewire surrounding cell populations to support aggressive tumor phenotypes by creating substances that support tumor growth. A wide variety of mechanisms are involved in the communication between malignant and surrounding cells, which can ultimately result in fast proliferation, metastasis, and treatment resistance or can act as a tumor suppressor (Liu et al., 2022). To fully comprehend these intricate interactions and their impact on tumor progression, advanced analytical techniques are necessary.

Single-cell RNA-sequencing (scRNA-seq) techniques can be used to unravel transcriptional differences in the very heterogeneous TME cell type composition transcriptome at single-cell resolution. This field has significantly advanced over the past 10 years since Tang et al. originally described the method of whole-transcriptome mRNA sequencing at single-cell resolution in 2009 (Tang et al., 2009) and is being particularly used in cancer research (Regev et al., 2017), scRNA-seq has become an essential tool for studies in the tumor microenvironment (Li et al., 2023). scRNA-seq detects the gene expression profile of each individual cell. By using this unbiased characterization, we are able to better understand the TME and identify the interactions that take place inside the tumor ecosystem (Wu et al., 2021). While scRNA-seq provides valuable insights into individual cell transcriptomes, integrating this data to understand cell-cell communication within the tumor microenvironment remains a challenge.

The study of cell‒cell communication and crosstalk between a tumor cell and other cell types in its microenvironment will enable new frontiers for effective cancer treatments (Wu et al., 2021). The issue of understanding how different cell types interact inside the TME to drive cancer and taking into account the many signals existing between and within different cell types is crucial in the field of cancer research (Chen et al., 2023; Bridges and Miller-Jensen, 2022; AlMusawi et al., 2021; Puram et al., 2017). To deeply investigate the TME, we require a model that can quantify both how signals are transferred between cells and how that information is processed within cells. Various methods exist for deriving these interaction, including CellPhoneDB (Efremova et al., 2020), SingleCellSignalR (Cabello-Aguilar et al., 2020), RobustCCC (Zhang et al., 2023), and Cellchat (Jin S. et al., 2020). These approaches leverage established databases that furnish valuable data concerning ligand-receptor interactions and regulatory networks involving transcription factors and target genes. By utilizing gene expression profiles, these methods construct multilayer networks, capturing diverse layers of molecular interactions. However, there is a lack of a comprehensive framework that can investigate cancer progression by examining inter-intra signaling networks. To address this gap, we propose a comprehensive framework that combines scRNA-seq data analysis with network-based approaches to investigate cancer progression through inter- and intra-cellular signaling networks.

**FIGURE 1:**
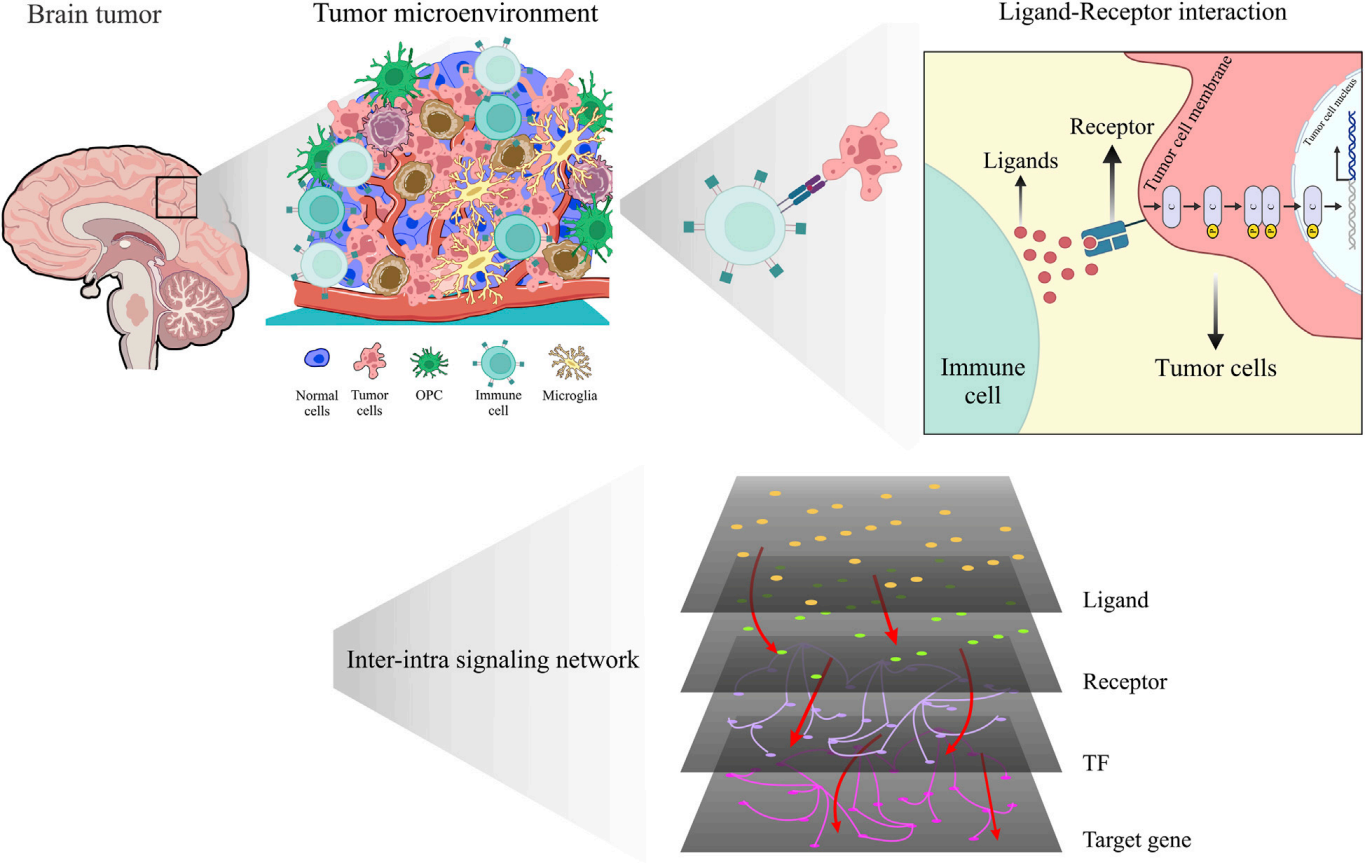
Unveiling the hidden dialog: revealing intricate interactions within the glioma microenvironment using scRNA-seq and inter-intra signaling networks.

The purpose of this study is to propose a pipeline for exploring molecular mechanisms in two stages of glioma cancer (grade III and grade IV glioma cells) by integrating scRNA-seq data and constructing a multilayer network that captures multiple layers of interactions, such as co-expression of ligand-receptor pairs across sender and receiver cells, gene regulatory networks in receiver cell, and signaling pathways, within the tumor ecosystem for each grade. We specifically focus on grade III and grade IV gliomas because these high-grade gliomas represent the most aggressive forms of the disease, with grade IV (glioblastoma) being the most lethal. Understanding the molecular differences between these two grades could provide crucial insights into the mechanisms of glioma progression. In Figure 1, we illustrate the implemented pipeline, which enables us to examine how communication between various cell types evolves in different glioma stages and to identify potential key drivers of glioma progression. The pipeline was applied to analyze glioma scRNA-seq data, revealing the communication patterns within the inter-intra signaling networks. In addition, our study incorporates complex network analysis, which includes topology and functional analysis, rewiring between two networks, and multiple centrality metrics, to analyze networks complex with different stages of cancer (grade III and grade IV). This analysis uncovers crucial signaling modules, significant ligand-receptor connections, and identifies the most significant ligand-receptor- TF axis. Furthermore, we employ pathway enrichment analysis (PEA) using the ReactomeFIVIz tool (Fabregat et al., 2018; Wu and Haw, 2017) to enhance our understanding of the TME and provided insight into the previously undiscovered connections between microscopic biological entities. This methodology employed to analyze glioma scRNA-seq data, allowed for a detailed exploration of the communication patterns within the networks. In our study, the enriched pathways exhibit quadruple signaling cascades, consisting of ligand-receptor-TF and target genes. Leveraging these findings, we selected several quadruples (a set of four genes, comprising a ligand, receptor, TF, and its corresponding target gene) as potential candidates for machine learning analysis to evaluate their efficacy in distinguishing grade III and grade IV samples within The Cancer Genome Atlas (TCGA) dataset. Furthermore, conducting differential network analysis between grade III and grade IV glioma cells allowed us to identify critical nodes and edges involved in the rewiring of molecular interactions. Lastly, we demonstrated the prognostic potential of this quadruples by predicting patient survival using Cox hazard models and Kaplan-Meier analysis. Additionally, we conducted a comparative analysis to assess the prognostic potential of the quadruple glioma biomarker in relation to other established biomarkers, demonstrating its better performance in predicting patient outcomes. These results showcase the significance of our approach in providing insights into the underlying mechanisms of glioma progression and its potential implications for clinical outcomes by inhibiting the connection between these quadruples.

## 2 Materials and methods

### 2.1 scRNA-seq data acquisition and quality control

The scRNA-seq data of glioma patients were collected from the Gene Expression Omnibus (GEO) database with accession number GSE89567 (Filbin et al., 2018). The dataset contained ten samples of glioblastoma patients from different stages (including seven samples from grade III, two samples from grade IV and one sample from grade II). For our analysis, patients in grades III and IV were taken into account. In total, 6,341 cells from publicly available datasets were downloaded and considered for downstream analyses.

The collected data were analyzed by using the “Seurat” package (Satija et al., 2015). Several filters were used to ensure that high-quality cells were retained for downstream analysis. Genes expressing <3 cells and cells expressing <50 genes were omitted. We accordingly excluded cells with a percentage of mitochondrial and External RNA Controls Consortium (ERCC) sequencing counts greater than 25%.

### 2.2 Normalization and dimension reduction

The R package Seurat v4.0.3 was used for all downstream analyses, which included normalization, dimensionality reduction, and Uniform Manifold Approximation and Projection (UMAP) visualization of the expression matrix (Satija et al., 2015). For the normalization step, the expression level of each gene within each cell was normalized by using the “NormalizeData” function of Seurat to eliminate any potential technical bias caused by differences in sequencing depths between cells. This allowed comparisons between different cells to be made (This function divides the expression level for each gene first divided by the total expression of all genes in each cell, subsequently multiplies the quotient by a scale factor of 10,000, and finally log-transforms the product.). After that, the top 2,000 highly differentially expressed genes were identified using the “FindVariableGenes” function for principal component analysis. For scaling and centering the expression levels of highly variable genes, “ScaleData” were used (Satija et al., 2015).

The “RunPCA” function was used to perform linear dimensionality reduction based on principal component analysis (PCA), which was based on the scaled and normalized expression data of variable genes. The first 20 PCs were used via “RunTSNE” (“RunUMAp”) functions to produce the t-SNE (UMAP) projections. The Harmony (Korsunsky et al., 2019) packages, widely recognized tools for scRNA-seq data integration and batch effects correction across datasets. Harmony was specifically chosen because of its ability to integrate scRNA-seq data from different cohorts while preserving biological variation and minimizing technical noise. In fact, In the PCA space, Harmony iteratively removes batch effects by clustering similar cells from different batches while maximizing batch diversity within each cluster.

### 2.3 Clustering and cell type assignment

Cells were clustered by applying the Seurat “FindClusters” function. This is a graph-based clustering method that creates a shared closest neighbor graph by calculating k-nearest neighbors based on PCA. It then optimizes a modularity function to find cell clusters. We applied a range of resolutions from 0.01 to 1 for the clustering analysis. After careful evaluation, we selected a resolution of 0.5 for our downstream analysis.

The cell types were identified according to the expression levels of marker genes that were obtained by the “FindAllMarkers” function of the Seurat package. With this function, genes that were differentially expressed between one cell cluster and the others were found. Cell types were assigned manually to clusters based on cell type marker expression specific to each cluster. Cell type markers were retrieved from the literature (Zhang et al., 2016; Dayanand and Olivia, 2018) and single-cell database (Franzén et al., 2019). In fact, clusters with cells that expressed marker genes for a specific cell type at the highest levels were assigned the corresponding cell type label. Based on a literature study, CCL3 and LAPTM5 can be cell-specific genes for immune cells, AGXT2L1 for astrocytes, SYT1 for neurons and MOG for oligodendrocytes. More importantly, EGFR can discriminate glioma cells with high sensitivity and specificity.

### 2.4 Construction of multilayer networks

The intercellular/intracellular signaling interactions (i.e., ligand‒receptor pairs, receptor-TF pathways, TF-target gene interaction) were constructed based on the scMLnet method (Cheng et al., 2021; Zhang et al., 2019). We tailored this method to suit our specific scenario. In our scenario, we considered immune cells as the sender cells and cancer cells as the receiving cells. The method involved several steps.

First, we collected ligand‒receptor pairing information from various databases and previous studies, similar to Zhang et al. (2019). This information served as the foundation for constructing the ligand‒receptor-mediated intercellular signaling network in our own context.

Next, we extracted the expression profiles of secretory ligand genes and cell surface receptor genes specifically in our immune and cancer cell types. To ensure robustness and minimize complexity, we focused only on highly expressed ligand genes in the immune cell type and cancer genes in the receiver cell type.

The constructed intracellular signaling network consisted of three layers: receptors, TFs, and target genes. To evaluate the significance of TF activation and regulatory relationships among TFs and their target genes, we employed Fisher’s exact test and LASSO regression models. Additionally, we connected the TF-target gene networks to the receptor layer by assessing the significance of the connected pathways, as outlined in Zhang et al. (2019).

To investigate the structural and functional characteristics of the TME networks, we utilized the igraph R package (Csardi, 2006). This enabled us to perform a comprehensive analysis of the interplay between the two TME networks in terms of their structural properties and functional behaviors.

### 2.5 Quadruple extraction and pathway enrichment analysis

By tracking the established signaling cascades from each ligand in immune cells to its appropriate receptor in cancer cells, as well as subsequent TFs and target genes, quadruples have been retrieved from multilayer signaling networks in each stage. The signaling of ligands that were cut off at the receptor or TF level was not taken into consideration. Then, the ReactomeFIVIz tool was used to conduct pathway enrichment analysis to identify biochemical pathways for signaling cascades.

### 2.6 Examining the power of quadruples via machine learning

Machine learning methods have been used to gain insight into the importance of the extracted quadruple signaling cascades. This stage included obtaining grade III and grade IV data from TCGA (Weinstein et al., 2013) and removing batch effect with Combat (Johnson et al., 2007) and Limma (Ritchie et al., 2015) package in R. After batch correction, patient samples were randomly divided into training, testing, and validation subsets. Next, extracted quadruples that only appear in the grade IV network were selected as key features for machine learning methods.

The collected datasets comprised 520 grade III samples and 172 grade IV glioma samples, resulting in a relatively imbalanced data distribution. This data imbalance is a prevalent issue in numerous classification tasks and poses a challenge in effectively training machine learning models. Notably, the models’ performance on the minority class is often crucial, yet it tends to be inadequately addressed by most techniques.

To mitigate this issue, we employed the synthetic minority oversampling technique (SMOTE) on the training datasets (Chawla et al., 2002). SMOTE was utilized to tackle the data imbalance problem by generating synthetic instances of the minority classes. This technique aims to augment the representation of the minority class until it achieves a balance with the majority classes. By introducing synthetic samples, the training dataset achieves a more balanced distribution, enabling machine learning models to learn effectively from both the majority and minority classes. The SMOTE algorithm was applied to the training datasets, resulting in the creation of synthetic samples for the grade IV class until it reached a balanced state with the grade III class.

Since there are numerous classification approaches, each with particular pros and cons, we had to choose the ones that were most appropriate for this problem. Several classifiers, including Decision tree (DT) (Quinlan, 1986), RF, K-Nearest Neighbors (Donaldson, 1967) (KNN), SVM, Linear Regression (LR) (Robert and Brown, 2004) and XGBoost (Chen and Guestrin, 2016), have been tested using the sci-kit learn python package (Barupal and Fiehn, 2019).

The classifiers specified above required a number of hyper-parameter tuning steps, all of which were carried out using the GridSearchCV technique. With the use of this strategy, the ideal configuration for the model that produces the best performance can be found by exhaustively exploring the hyper-parameter space.

In this study, to ensure a complete evaluation of the prediction models by training and testing them on different random combinations of data, 100 iterations of five-fold cross-validation tests using different random seeds were carried out. Then, among the most often used metrics for evaluating a machine learning model, accuracy, recall, precision, and F1 scores were obtained for each fold, and the average of these metrics across all folds was calculated.

### 2.7 Construction of the prognostic model based on the selected quadruple

To investigate the prognostic correlation of the specific quadruple, we performed a series of analyses on gene expression data and patient survival information from glioma high-throughput sequencing data. We obtained gene expression profiles and survival data from the TCGA database.

The TCGA samples of glioma patients were randomly divided into training and test sub datasets using a variety of sample ratios (60%, 70%, 80%, and 90%) relative to the total sample size (N = 702). We first applied univariate Cox regression analysis to identify the prognostic genes in the training cohort. This analysis allowed us to identify genes whose expression levels were significantly associated with patient survival. We then used multivariate Cox regression analysis (Cox, 1972) with Efron approximation to establish a risk score model for predicting patient survival. The model was expressed as a linear combination of the expression levels of the prognostic genes and their corresponding coefficients (beta values):

$$\text{risk score}=\beta_{\text{gene}1}\times {\text{Expression}}_{\text{gene}1}+\beta_{\text{gene}2}\times {\text{Expression}}_{\text{gene}2}+\beta_{\text{gene}3}\times {\text{Expression}}_{\text{gene}3}+\beta_{\text{gene}4}\times {\text{Expression}}_{\text{gene}4}$$

To evaluate the performance of the risk score model, we generated ROC curves (receiver operating characteristic) and Kaplan‒Meier (Kaplan and Meier, 1958) survival curves for both the training and validation cohorts. ROC curves were used to assess the sensitivity and specificity of the model in predicting patient survival. Kaplan‒Meier survival curves were used to estimate the survival probability over time for patients with high and low risk scores.

### 2.8 Comparisons with other related signatures and other methods

To evaluate the prognostic accuracy of the selected quadruple in predicting the overall survival of glioma patients, we performed a comparative analysis with other established risk signatures using the same data for the training and testing procedures. Specifically, we compared the selected quadruple with the following risk signatures:

1) The multilayer network biomarker obtained from Zhang et al. study: This signature was derived from a previous study and has been shown to be a strong predictor of glioma patient survival (Cheng et al., 2021).
2) EGFR and ERBB2 signatures: These signatures are based on the expression levels of two genes that have been implicated in glioma progression and are commonly used as prognostic biomarkers.
3) A signature developed by Cheng et al. was developed using a machine learning approach and has been shown to accurately predict the survival of glioma patients (Cheng et al., 2016).

To compare the prognostic accuracy of these signatures, we calculated the AUC of the ROC curve for each signature.

## 3 Results

### 3.1 Quality control and normalization

**FIGURE 2:**
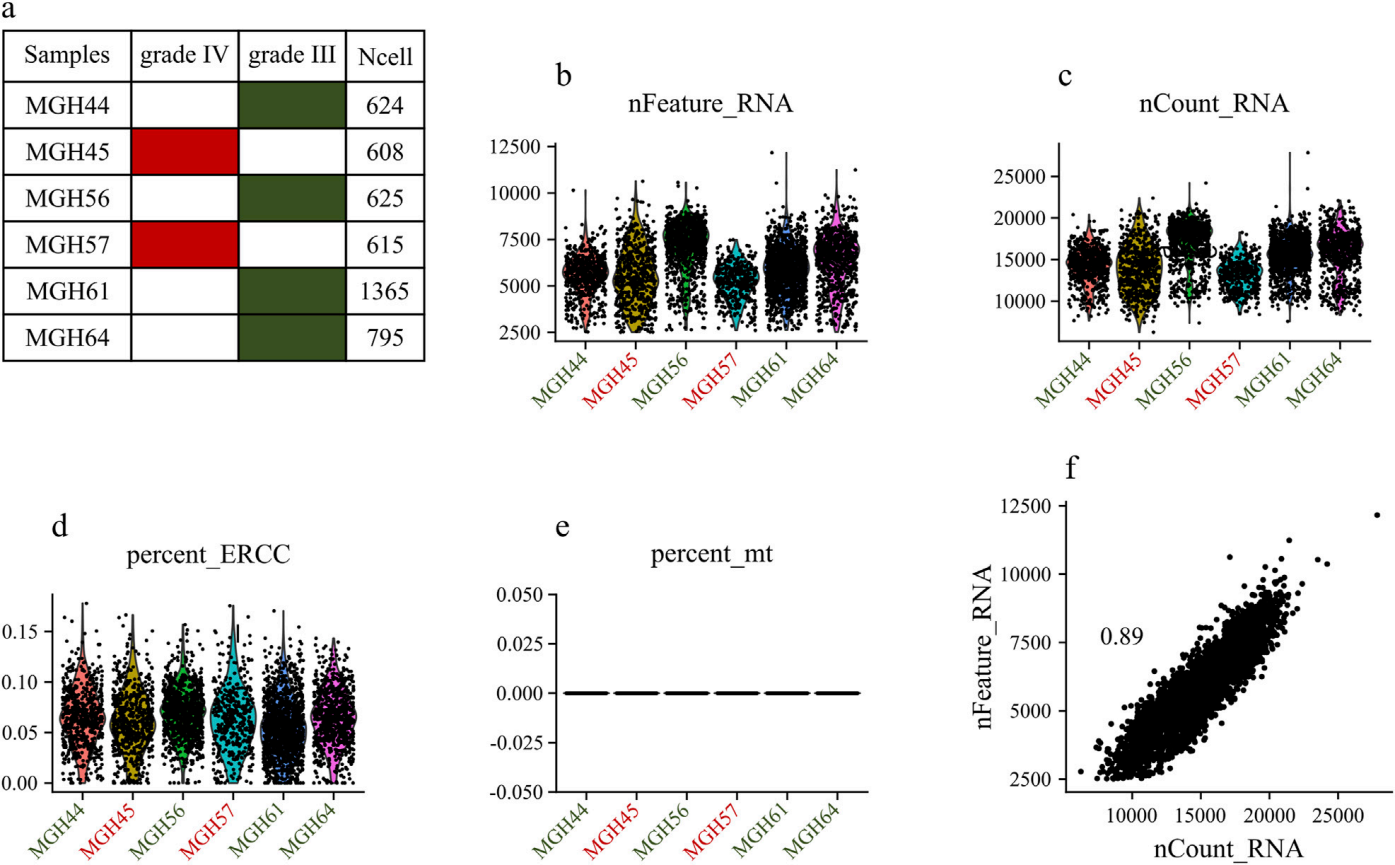
Exploring Single-Cell Data: **(A)** Statistics of analyzed samples. **(B)** Detected gene profile per sample. **(C)** Total gene count per sample. **(D)** Mitochondrial gene percentage in each sample. **(E)** ERCC gene percentage in each sample. **(F)** Correlation between the numbers of sequenced genes and sequencing depth in each sample.

The dataset GSE89567 contains expression data from 6,341 cells collected from six individuals, of which four were diagnosed with grade III tumors, and two with grade IV tumors. Figures 2A–E displays general statistics of samples, the total number of genes expressed in each sample, the numbers of genes expressed in each sample, and the percentages of mitochondrial and ERCC sequencing count in each sample. As shown in Figure 2D, the percentages of mitochondrial sequencing samples were relatively low. This may be because relatively few apoptotic or lysed cells were produced during the single-cell sorting procedure. Figure 2F shows the correlation between the number of sequenced genes and the depth of sequencing in samples. Stringent quality control criteria, as detailed in the Section 2, were applied to remove low-quality cells. After QC filtering, we retained 6,241 high-quality cells. To account for variations in sequencing depth per cell, filtered raw read counts were normalized. We also scaled the data and performed regression analysis against gene count expression.

**FIGURE 3:**
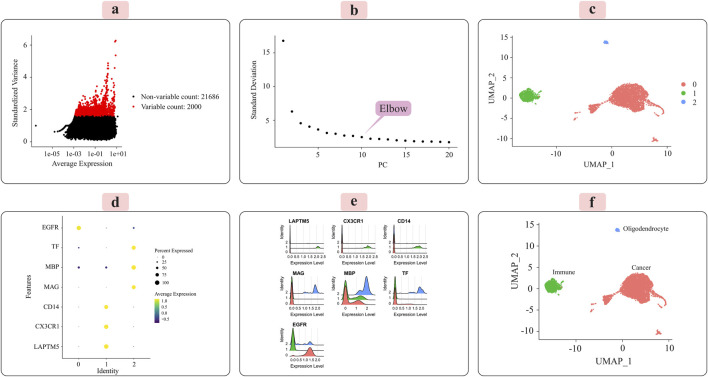
Presents the key results from applying the Seurat analytical pipeline to the normalized single-cell RNA-sequencing data for grade III tumors. **(A)** The distribution of the 2000 most highly variable genes across cells is shown. **(B)** Principal component analysis was employed for dimensionality reduction prior to clustering. **(C)** Cells were clustered in an unsupervised manner according to their transcriptomic profiles. **(D)** A dot plot displays the expression levels of marker genes characteristic of immune cells, oligodendrocytes, and cancer cells across the identified clusters. **(E)** Ridge plots further visualize the expression patterns of each marker gene across clusters. **(F)** Finally, the UMAP plot provides a low-dimensional embedding, with clusters annotated as their putative cell types based on the observed marker gene expression signatures. The distinct cell populations are well-separated in this visualization.

**FIGURE 4:**
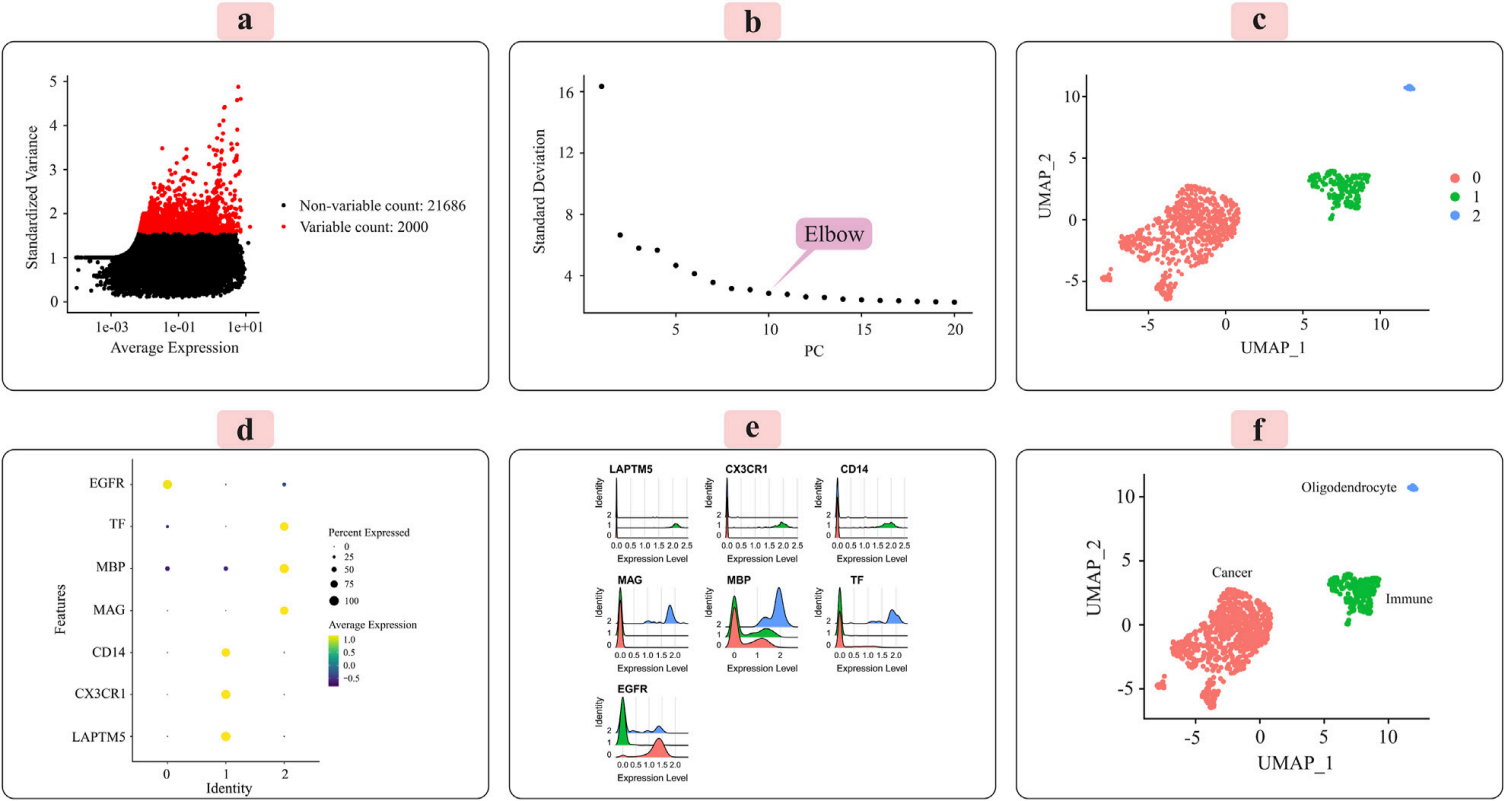
Illustrates the key results obtained from applying the Seurat analysis pipeline to the normalized single-cell RNA-sequencing data for grade IV tumors. **(A)** The distribution of the 2000 most highly variable genes across cells is depicted. **(B)** Principal component analysis was used for dimensionality reduction prior to clustering. **(C)** Cells were clustered in an unsupervised manner based on their transcriptomic profiles. **(D)** A dot plot displays the expression patterns of marker genes characteristic of immune cells, oligodendrocytes, and cancer cells across the identified clusters. **(E)** Ridge plots further visualize the expression of each marker gene across clusters. **(F)** Finally, clusters were annotated with their putative cell types based on the marker gene expression patterns, with the UMAP plot providing a low-dimensional embedding separating the distinct cell populations.

The distribution of the 2,000 features exhibiting the most significant cell-to-cell variation is depicted in Figure 3A for the grade III dataset and Figure 4A for the grade IV dataset. Numerous prior studies have shown that concentrating subsequent analyses on these highly variable genes effectively captures the underlying biological signals present in single-cell data (Csardi, 2006).

Principal component analysis (PCA) was applied to the scaled data to facilitate downstream classification and other analyses. The goal was to determine the optimal number of principal components (PCs) required to capture most of the variance within the datasets. Elbow plots, shown in Figure 3C for grade III and Figure 4C for grade IV, aided in visualizing and identifying the appropriate number of PCs to retain.

### 3.2 Unsupervised clustering and cell-type annotation

We used the Seurat package (Satija et al., 2015) to perform unsupervised cell sample clustering based on single-cell gene expression data (see Section 2). Within the grade III dataset, there were a total of 5,057 cells, and within the grade IV dataset, 1,087 cells were distinctly divided into three primary clusters, as illustrated in Figures 3C, 4C. These clusters were composed of 3,335 cells, 310 cells, and 57 cells for grade III and 766 cells, 235 cells, and 22 cells for grade IV.

Manual annotation of cell clusters was performed by leveraging literature information and expression patterns of known marker genes for common cell types. Figures 3D, 4D depict the expression levels of these marker genes across the identified clusters for grade III and grade IV tumors, respectively. Genes such as MBP, MAG, and TF exhibited preferential expression in cluster 2, suggesting these cells are oligodendrocytes. Cluster 1 highly expressed immune cell markers like CD14, LAPTM5, and CX3CR1, indicating an immune cell population. Cancer cell markers, including EGFR, were enriched in cluster 0, suggesting these are the cancer cells. Ridge plots (Figures 3E, 4E) further illustrate the expression patterns of these markers across clusters. The UMAP visualizations in Figures 3F, 4F provide a comprehensive overview, with the distinct annotated cell types clearly separated in the low-dimensional embedding for both grade III and grade IV datasets.

We performed functional enrichment analysis of the marker genes in each cell type to validate the cell type inference. Cluster 1 cells were significantly enriched primarily in inflammatory response and immune response functions (Supplementary Figure S1), supporting the inference of cluster 1 cells as immune cells. Meanwhile, cluster 2 cells were significantly enriched in oligodendrocyte differentiation, oligodendrocyte development, myelination, etc. (Supplementary Figure S2). These biological functions support the identification of cluster 2 cells as oligodendrocytes, a type of neuroglia that provides support and insulation to axons in the central nervous system.

Additionally, Supplementary Figure S3 presents the heatmap representation of each most significant marker for each cluster as additional support for the cell type annotation part. When combined together, we identified that the cells in cluster 0 were cancer cells. The cells in cluster 1 were immune cells, and the cells in cluster 2 were oligodendrocytes. As a result, we were able to identify the different types of cells in our scRNA-seq data, which served as the foundation for subsequent multilayer intercellular/intracellular communication network construction.

### 3.3 Multilayer signaling network investigation

**FIGURE 5:**
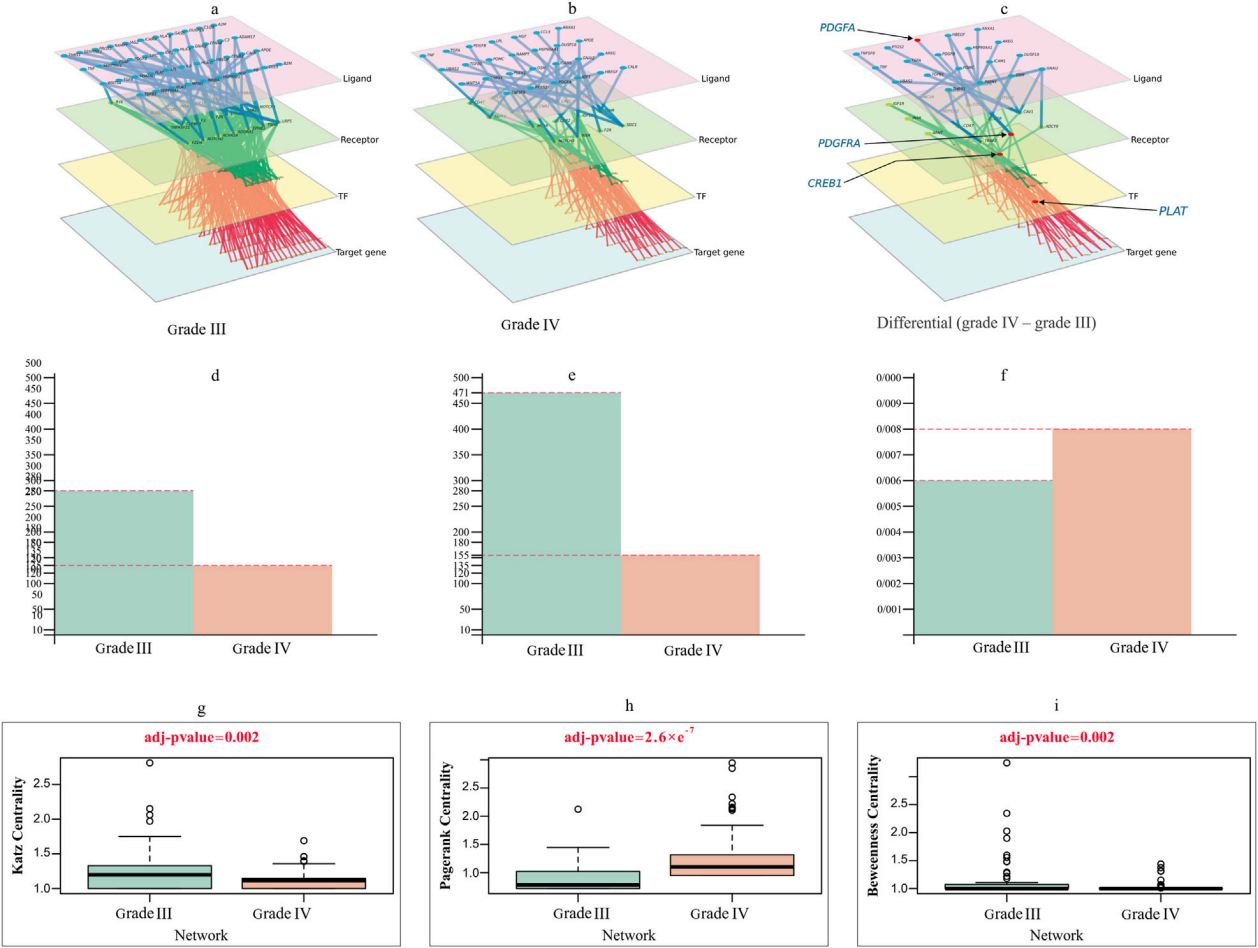
Multilayer intercellular/intracellular signaling network of tumor cells activated by immune cells in **(A)** grade III and **(B)** grade IV. **(C)** Differential network only exists in grade IV, highlighting four crucial genes forming the important quadruple for the prediction task. The nodes in different layers represent ligands (pink), receptors (green), TFs (yellow) or target genes (blue). **(D–F)** Characterization of the difference between grade III and grade IV networks in **(D)** degree, **(E)** edge and **(F)** degree distribution. **(G–I)** Violin plots comparing the differences in **(G)** Katz centrality **(H)** PageRank and **(I)** Betweenness centrality between grade III and grade IV networks, with *t*-test adjusted *p* values of 0.002, 2.6e^−7^ and 0.002.

The annotated output of the cell type analysis for each cluster in the glioma samples was used as the input of scMLnet (Cheng et al., 2021; Zhang et al., 2019). Immune cells are abundant cell types in the microenvironment associated with tumors and play important roles in tumor growth and drug resistance. Therefore, we studied the interactions between glioma cells (as receiver cells) and immune cells (as sending cells) for two stages separately. Inter/intracellular multilayer networks for each stage are illustrated in Figures 5A,B. These multilayer signaling networks consist of four layers: ligand layer, receptor layer, TF layer, and target gene layer. In each layer, by moving from grade III to grade IV glioma, the number of interactions decreases. Specifically, we observed 471 interactions in grade III, whereas grade IV stage exhibited a considerably lower count of only 155 interactions. This occurred as a result of cancer cells’ desire to be more independent and their dislike of receiving connections from other cell types.

The primary intercellular signaling pathways between glioma cells and immune cells and the intracellular signaling networks of tumor cells in response to the stimulus of ligands released by immune cells are used to study the interactions between glioma cells and immune cells in two grades. The intercellular and intracellular networks were constructed using the highly expressed genes, which included 43 immune-secreted ligand genes, 29 receptor genes, 48 TFs and 153 target genes in tumor cells for grade III and 27 immune-secreted ligand genes, 18 receptor genes, 18 TFs and 70 target genes in tumor cells for grade IV (Figures 5A,B). Figure 5C depicts the differential network, which emphasizes the differences between the two stages being referred to. This network showcases the contrast between the two stages and provides a visual representation of their contrasting relationships.

We examined the topological difference between the grade III and grade IV multilayer networks (MLNs). As shown in Figures 5D–F, the node degree of the grade III network was significantly larger than that of the grade IV network. The number of nodes and edges in the grade IV network is fewer than that in the grade III network. Figures 5G–I illustrates how different centrality measurements and criteria have changed over stages. These results evidently show that some nodes in one network were highly important, but they do not play a significant role in the other.

Considering degree centrality, the MYC gene is a crucial TF in grade IV. This gene plays a crucial role in regulating the cell cycle and metabolism. Furthermore, MYC boosts the ability of glioma stem-like tumor/tumorsphere cells to self-renew and increases their tumorigenic potential. MYC is therefore a compelling therapeutic target in GBM. Despite considerable attempts, direct inhibition of the MYC TF has remained a challenge. Hence, indirect strategies should be used. A strategy can be proposed using MLN. In this strategy, we identified the upstream genes of this TF, and instead of directly suppressing MYC, we can prevent the binding of its receptor or, at a higher level, the production of the relevant ligand in immune cells (Tateishi et al., 2016).

ETS1 has a high betweenness centrality in the grade III network and is crucial for the network’s ability to transit information between layers, but it loses this position and function in the grade IV network. This could be because ETS1 is critical for the transcription of key genes involved in invasion, migration, and proliferation in GBM. Tang et al. identified ETS1 as a critical regulator of tumor angiogenesis and vascular abnormalities in GBM (Jin L. et al., 2020; Tang et al., 2021).

Another crucial gene in GBM MLNs is the Cav-1 receptor, but this receptor is not active in grade III. Due to its function in metastasis, invasion, and progression, this receptor is suggested as a therapeutic target in GBM treatment. This receptor appears to play a pivotal role in adjusting immune-tumor interactions by promoting tumor growth, metastasis, resistance to therapy, and cell survival (Parat and Riggins, 2012; Ketteler and Klein, 2018).

### 3.4 Quadruple extraction and pathway enrichment analysis (PEA)

Next, we focused on multilayer signaling networks to find quadruple signaling cascades composed of ligands, receptors, TFs, and target genes.

Following the signaling cascades that were established from each ligand to its target genes, 2,290 and 349 quadruple signaling cascades were discovered for grade III and grade IV, respectively. Supplementary Table S1 contains more information about these cascades.

Then, PEA was conducted to find biochemical pathways for signaling cascades through the ReactomeFIVIz tool. The results show that the 349 quadruple cascades originate from 21 ligands for grade IV. Among them, numerous signaling cascades related to 14 ligands were significantly involved in 13 biochemical sub-pathways in cancer (*FDR* < 0.05). These sub-pathways are a subset of three main biochemical pathways, including signal transduction, immune system, and gene expression Supplementary Table S2 displays these pathways. More investigation of Supplementary Table S2 indicates that the majority of signaling cascades are engaged in the sub-pathway known as signaling by receptor tyrosine kinases (RTKs).

Signaling by RTK is an important biochemical pathway in cancer. Aberrant signaling by RTKs is a commonly observed factor in cancer, and many genes related to this pathway have been studied as potential therapeutic targets for cancer-related malignancies (Fauvel and Yasri, 2014). RTKs are membrane receptors that are of great clinical interest due to their role in diseases, especially cancer. Various mechanisms of RTK dysregulation are known to lead to the occurrence of multiple cancers. Consequently, RTKs have emerged as an important class of targeted cancer therapeutics over the past 20 years. Currently, a large number of drugs based on small molecules with the role of tyrosine kinase inhibitors have been developed and clinically approved for several cancers. However, many RTK inhibitor-based therapies lead to acquired resistance and tumor recurrence. Therefore, it is crucial that new technologies be employed to find various types of RTK inhibitors to target both the receptors and the critical cellular factors that play a pivotal role in the RTK signaling cascade (Saraon et al., 2021). Evidence based on the literature shows that the signaling pathways regulated by RTKs are considered drug targets in glioblastoma (Lemmon et al., 2014; Qin et al., 2021). Additionally, it has been demonstrated that one of the recognized oncogenic drivers in GBM is epidermal growth factor receptor (EGFR), which is frequently the major RTK. Indeed, GBM has the highest frequency of alterations in the EGF/EGFR pathway across 33 types of human cancer (Long et al., 2023).

To identify relevant biochemical pathways for grade III, 2,290 quadruple cascades initiated from 43 ligands were investigated. The results significantly support the involvement of 14 biochemical sub-pathways in multiple signaling cascades initiated by 11 ligands (*FDR* < 0.05). All these pathways are important in carcinogenesis and are subsets of the three main biochemical pathways: signal transduction, gene expression, and developmental biology. The result of this part is shown in Supplementary Table S3.

Additionally we performed a Gene Set Enrichment Analysis (GSEA) and utilized the DisGeNET database (available at disgenet.org) (Piñero et al., 2017), which is a comprehensive platform that integrates genetic, clinical, and functional data related to gene-disease associations, to investigate the enrichment of genes associated with tumor progression, particularly in GBM.

Our analysis identified *PDGFRA* and *PDGFA*—both of which are findings from our investigation—as key genes significantly enriched in pathways that are critical for malignant glioma processes. These genes are involved in crucial signaling pathways such as R-HSA-162582 and R-HSA-109582, both of which regulate cell proliferation, apoptosis, and tumorigenesis—core mechanisms driving the progression of high-grade gliomas like GBM.

Additionally, the GDA (Gene-Disease Association) scores for these genes, particularly *PDGFRA* with a score of 0.75, provide strong evidence linking them to cellular proliferation and tumor growth. This high GDA score reflects robust validation from multiple biological and clinical sources, underscoring the essential role these genes play in the progression of GBM. The results of this analysis are presented in Supplementary Table S4.

### 3.5 Performance evaluation of the quadruple signature model

A key objective of this study was to identify the critical quadruples (ligand-receptor-transcription factor-target gene) that significantly discriminate between grade III and grade IV gliomas. Two criteria were employed to discern grade IV-specific signaling cascades: 1) Quadruples unique to grade IV compared to the grade III signaling cascades (Supplementary Table S1), and 2) Confirmation of the selected quadruples in the enriched list obtained via ReactomeFIViz analysis (Supplementary Table S2). Adhering to these stringent criteria enabled the identification of pivotal quadruples that hold promise for elucidating the molecular underpinnings underlying the transition from grade III to grade IV. Among these, one quadruple (*PDGFA-PDGFRA-CREB1-PLAT*) effectively differentiated the two grades.

**FIGURE 6:**
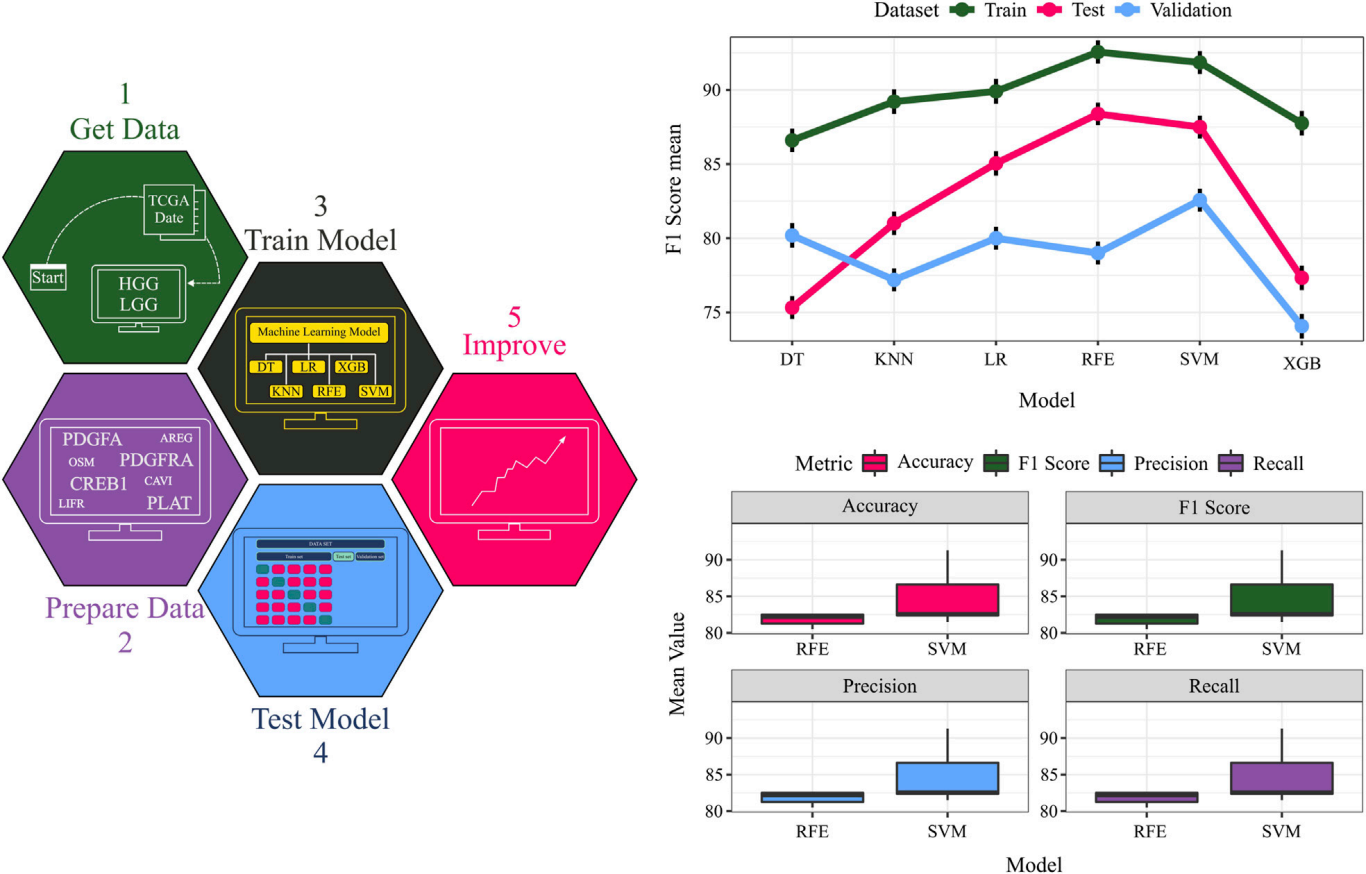
The TCGA dataset serves as input data. Quadruples detected in the data are considered crucial features, and their expressions are extracted from the input data. The data is split into training, testing, and validation sets, and diverse classifiers are employed for model training on the training data. Model performance is evaluated on the test and validation data. A comparative assessment of different models is conducted on training, testing, and validation sets. The F1 score values for each dataset using various models provide an overall performance measure.

Model performance was rigorously evaluated using 100 iterations of stratified five-fold cross-validation (CV). To ensure an unbiased assessment, datasets were shuffled with different random seeds prior to the train-test split in each CV iteration. Furthermore, the model’s generalization capability was tested on an untouched 20% validation set held out from the training procedure. The machine learning workflow and CV results are presented in Figure 6 and Table 1.

**TABLE 1:** A comprehensive overview of the model’s performance by presenting the average values of four key metrics: accuracy, precision, recall, and F1-score. These metrics are calculated for both the validation set and the test set.

	Test				Validation			
Models	Accuracy	Precision	Recall	F1 Score	Accuracy	Precision	Recall	F1 Score
KNN	81.01	72.73	91.43	82.01	77.19	75.86	78.57	77.19
DT	75.32	69.05	82.86	75.32	77.19	75.86	757	80.19
SVM	82.05	74.42	94.29	87.5	83.57	81.57	88.57	84.57
RF	82.05	74.56	91.43	87.05	80	81.48	88.57	80.12
XGB	77.33	72.5	82.86	77.33	74.07	76.92	78.57	74.07
LR	82.05	74.36	88.86	85.33	80	81.48	78.57	80.07

To identify the optimal classifier, support vector machines (SVM) and random forests (RF) were benchmarked against other algorithms, exhibiting superior performance across all datasets. Notably, the ability to discriminate between grades using only four features (ligand, receptor, transcription factor, and target gene) underscores the potential of complex network analysis in biology. While these four genes (*PDGFA, PDGFRA, CREB1, and PLAT*) have been studied individually, our findings highlight their collective significance in glioma. Additionally, the physical interaction between the identified ligand (*PDGFA*) and receptor (*PDGFRA*) was validated using the STRING database. Below, we explain the significance of these four features in grade IV biology.

Amplification and mutation of platelet-derived growth factor receptor A (*PDGFRA*) are frequently observed in high-grade gliomas, particularly glioblastoma, and correlate with a more aggressive phenotype. *PDGFRA*, a RTK, activates crucial downstream signaling pathways such as the phosphoinositide 3-kinase/protein kinase B (PI3K/AKT) and rat sarcoma/mitogen-activated protein kinase (RAS/MAPK) pathways, which promote cell proliferation, survival, and migration. Platelet-derived growth factor A (*PDGFA*) acts as a ligand for *PDGFRA* and stimulates its activity (Pandey et al., 2023). A physical connection has been reported between *PDGFA* and *PDGFRA* genes based on the string database. Inhibition of *PDGFRA* has demonstrated reduced tumor growth and improved survival in preclinical glioblastoma models (Yang et al., 2023).

Cyclic AMP-responsive element-binding protein 1 (*CREB1*) is a TF commonly overexpressed in high-grade gliomas. *CREB1* regulates the expression of genes involved in cell proliferation, survival, and invasion, including *PDGFA* and *PDGFRA*. Furthermore, *CREB1* interacts with the EGFR pathway and contributes to resistance against EGFR inhibitors. Inhibition of *CREB1* has displayed reduced tumor growth and invasion in preclinical glioblastoma models (Qian et al., 2015; Zhang et al., 2020). In addition, our findings align with previous research by Gammoh et al., highlighting the intricate relationship between *PDGFRA* and *CREB1*. These results underscore the significant regulatory role of *CREB* in modulating *PDGFRA* expression, providing further insight into the molecular mechanisms governing this regulatory pathway in our cellular model.

The plasminogen activator, tissue-type (*PLAT*) gene encodes tissue plasminogen activator (tPA), a protein involved in the breakdown of blood clots. In gliomas, tPA promotes invasion and angiogenesis by activating the extracellular matrix and releasing growth factors. High levels of tPA expression have been associated with a poor prognosis in glioblastoma patients (Sawaya et al., 1991). Inhibition of *PLAT* may have a great effect on reducing glioblastoma cell invasion in preclinical studies.

Overall, these molecules are believed to play critical roles in glioblastoma progression and represent potential therapeutic targets for the treatment of this disease.

### 3.6 Validation of the quadruple signature on TCGA dataset

To further assess the robustness and generalizability of the identified quadruple (*PDGFA*-*PDGFRA-CREB1-PLAT*), its predictive performance was evaluated on an independent validation set comprising 20% of the TCGA data, which was held out and not used during any stage of the training process. This rigorous validation on unseen data provides an unbiased estimate of the model’s real-world performance.

The strong predictive power exhibited by this single quadruple on the validation set underscores its potential as a robust biomarker for distinguishing grade III and grade IV gliomas. These findings not only corroborate the importance of the *PDGFA-PDGFRA-CREB1-PLAT* signaling axis in glioma progression but also demonstrate the efficacy of our network-based approach in pinpointing critical molecular determinants of disease states. The predictive performance of the top-performing models on the validation set, as measured by accuracy, precision, recall, and F1 score, is presented in Table 1 and Figure 6.

### 3.7 Validation of the quadruple signature on an independent dataset

**FIGURE 7:**
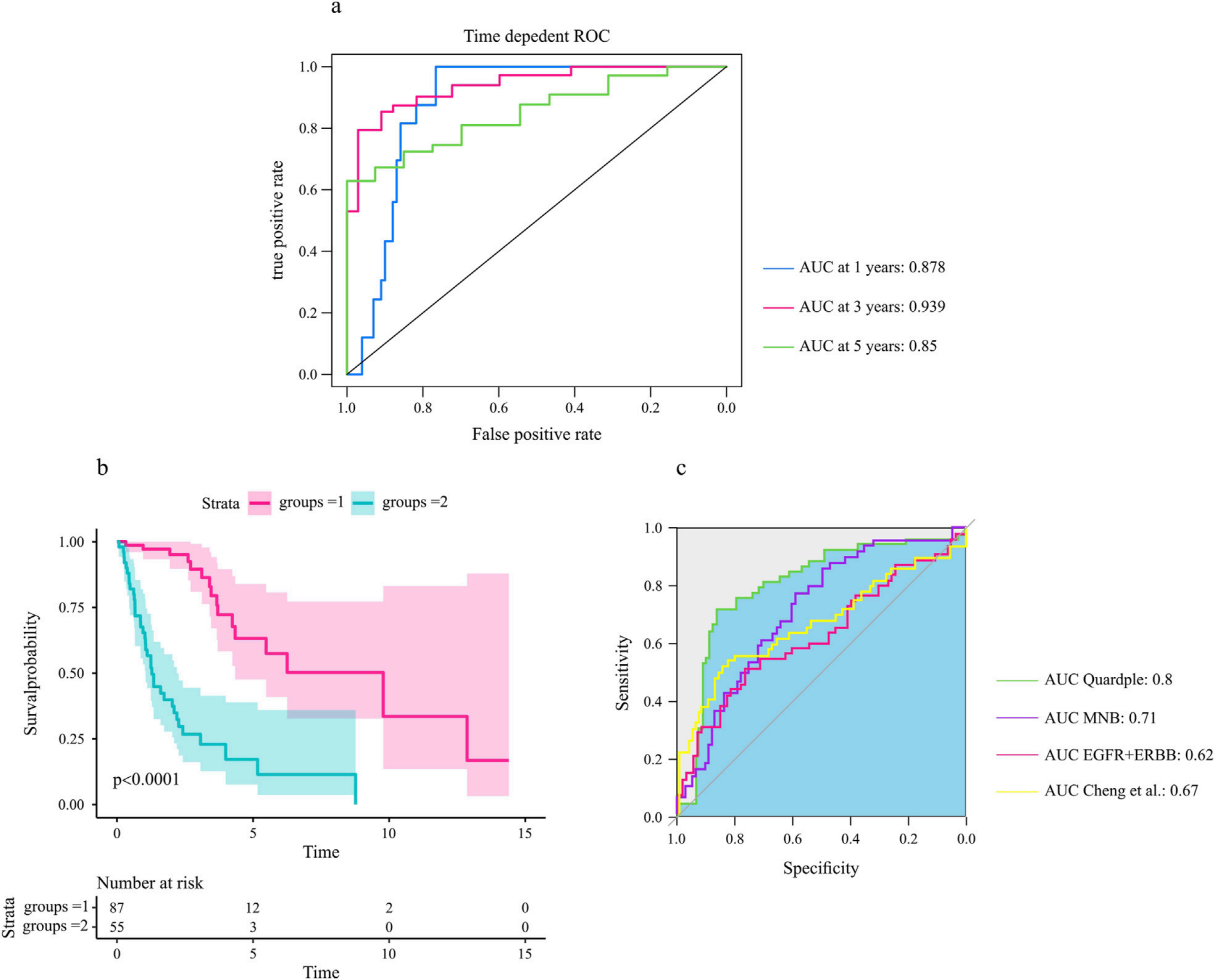
The selected quadruple predicted the prognosis of glioma patients. **(A)** The area under the ROC curves for 1-year, 3-year, and 5-year overall survival in the test cohort. **(B)** The four identified genes were associated with the survival probability of glioma patients (N = 172). A log rank test was used to assess the significance between two curves, with a *p*-value less than 0.0001. **(C)** Comparative analysis of the selected quadruple with other prognostic biomarkers (Section 2).

Our hypothesis was that the genes included in the selected quadruple genes would be associated with survival outcomes in glioma patients. We used the univariate Cox proportional hazards model to assess the association of each gene in the selected quadruple with the prognosis of glioma patients. The selected quadruple contained one ligand gene, one receptor gene, one TF, and its target gene, with corresponding coefficients of (−0.06759852, 0.08573037, −0.84328532, 0.82401982). Based on the risk score calculated by the prognostic model, the ROC curves and Kaplan‒Meier survival curves for the training and validation cohorts are shown in Figures 7A,B.

The analysis of the prognostic accuracy of the selected quadruple model revealed promising results for predicting overall survival in GBM patients. Specifically, the areas under the ROC curves for 1-year, 3-year, and 5-year overall survival in the training cohort were 0.88, 0.955 and 0.871, respectively. The corresponding AUCs in the test cohort were 0.87, 0.93 and 0.85, respectively (Figure 7A).

Furthermore, the Kaplan‒Meier survival analysis showed significant differences in survival probability between patients with high and low risk scores, as evidenced by *p* values of 0.0001 for the test cohorts (Figure 7B). These findings indicate that the selected quadruple-based prognostic model is capable of accurately stratifying patients according to their survival outcomes.

Overall, the results suggest that the selected quadruple model has an excellent prognostic ability for gradeIII and gradeIV glioma and could serve as a valuable procedure for predicting patient survival.

To assess the relative prognostic accuracy of the selected quadruple model compared to other established biomarkers, we performed a comprehensive comparative analysis using our dataset. Specifically, we calculated and compared the area under the ROC curves of each biomarker for predicting overall survival in glioma patients (Figure 7C).

Our results revealed that the selected quadruple model exhibited superior prognostic accuracy compared to other signatures. These findings suggest that the selected quadruple model could serve as a reliable prognostic tool for stratifying glioma patients according to their survival outcomes.

## 4 Discussion

Gliomas, one of the most lethal primary brain cancers, pose a significant challenge to current treatment modalities such as surgery, radiotherapy, and chemotherapy, which have only modestly improved patient survival rates (Ostrom et al., 2013). Consequently, there is a pressing need for more effective therapeutic strategies and drug targets. Investigating the molecular mechanisms underlying the disease’s progression from low-risk to aggressive stages could facilitate the development of improved therapies (Stuart and Satija, 2019).

A critical aspect of understanding cancer progression is elucidating cell‒cell communication in the TME, particularly via ligand‒receptor complexes that differentiate grade IV cellular states from grade III (Quail and Joyce, 2013). Systematic reconstruction of the TME, achieved by modeling intercellular signaling pathways and intracellular gene regulatory networks, can provide valuable insights into cancer progression and aid in designing more effective therapeutic methods (Peng et al., 2022). With the advancement of scRNA-seq technologies, multilayer signaling networks and network analysis tools are becoming increasingly important in studying cancer progression (Lah et al., 2020).

To illustrate our approach, we used scRNA-seq transcriptome data from glioma patients (including grade III and grade IV patients). To examine connections between tumor cells and their surroundings along the progression road, we constructed two multilayer intercellular/intracellular signaling networks. In these networks, immune cells play the sender cell role, and cancer cells play the receiver cell role. Then, differential networks between these two stages are used to identify quadruples that play important roles in transferring from grade III to grade IV.

In this study, we employed scRNA-seq data to model and reconstruct multilayer signaling networks that underlie glioma progression from grade III to grade IV. These networks involve the transmission of information from ligand‒receptor interactions between cells to intracellular TFs and subsequently to target genes. We utilized scRNA-seq transcriptome data from glioma patients, including those with grade III and grade IV glioma, to construct two multilayer intercellular/intracellular signaling networks that examined the connections between tumor cells and their surroundings during progression. In these networks, immune cells acted as sender cells, and cancer cells acted as receiver cells. Differential networks between these two stages were analyzed to identify key quadruples involved in the transition from grade III to grade IV glioma.

Our study illuminates previously uncharted aspects of glioma progression, offering novel biological insights that underscore the complexity of tumor microenvironment interactions:

1. As demonstrated in Figures 5D,E, our findings reveal significant differences between these two groups, with grade IV networks exhibiting a notable decrease in connectivity with the TME. The observed behavior of grade IV cancer cells suggests a preference for independent activity, characterized by detachment from the extracellular matrix and enhanced motility, which in turn facilitates the creation of metastases in nearby tissues. These results are consistent with previous studies that have shown a strong association between tumor grade and metastatic potential (Lah et al., 2020). Our findings highlight the importance of understanding the underlying mechanisms of tumor progression and the potential impact of network disruptions on disease outcomes.
2. By comparing different node centrality measures in the two networks, we identified nodes that either lost or gained significance during progression (Figures 5F–H). Our findings provide valuable insights into the changes in network structure and suggest the presence of key nodes that play critical roles in tumor progression. The identification of these nodes can have important implications for the development of new therapeutic strategies aimed at inhibiting or stimulating crucial interactions that drive tumor progression. For example, inhibiting interactions associated with nodes that show a gain in significance during progression may represent a promising therapeutic approach to halt tumor growth and metastasis. Conversely, stimulating interactions associated with nodes that show a loss of significance may enhance anti-tumor immunity or promote tumor regression.
3. The identification of a quadruple mentioned in the Section 3 (*PDGFA–PDGFRA–CREB1*–*PLAT*) with the ability to effectively differentiate between grade III and grade IV states has significant implications for glioma patients. We developed a machine learning-based model capable of discriminating between grade III and grade IV patients using only this quadruple (four genes). These genes play crucial roles in glioblastoma progression and metastasis and are involved in key cellular processes such as cell growth, division, differentiation, and development (Pandey et al., 2023; Yang et al., 2023; Qian et al., 2015). Our study highlights the collective importance of four specific genes in glioma patients, suggesting that they may play a critical role in glioma development and progression. Incorporating these identified genes as significant features in a machine learning model offers the opportunity to attenuate the signaling generated by these genes at various levels. For instance, one approach could involve disrupting the ligand-receptor interaction by designing a ligand similar to the desired receptor’s natural ligand.

The discovery of this quadruple (*PDGFA–PDGFRA–CREB1–PLAT*), capable of distinguishing between grade III and grade IV gliomas, holds substantial implications for patients affected by these tumors. Activation of the Signaling by Receptor Tyrosine Kinases pathway by these four genes underscores their pivotal role in regulating cellular growth and proliferation, as delineated in the results. This research provides insights into the potential application of targeted interventions aimed at disrupting this signaling pathway and interrupting the molecular mechanisms driving metastasis.

4. We successfully demonstrated the predictive value of the identified quadruple in estimating patient survival using Cox hazard models and Kaplan-Meier analysis. These genes exhibited significant prognostic and predictive capabilities in clinical datasets of TCGA databases for glioma patients. Notably, our findings underscore the superiority of biomarkers that encompass cell-cell interaction processes over traditional biomarkers that solely focus on tumor cells. This emphasizes the crucial role of comprehending the intricate interplay between tumor cells and their microenvironment in prognosticating patient outcomes. It is important to note that while the identified biomarkers were derived from single-cell analysis and multilayer networks, their accuracy was validated on bulk data. This validation process underscores the robustness and potential clinical applicability of these biomarkers in accurately predicting patient survival in the context of glioblastoma.
5. The model and framework employed in this study, which effectively identified significant genes associated with disease progression between grade III and grade IV, introduce a novel approach applicable to comparative investigations focusing on the TME. This framework holds potential for studying the TME in the context of drug resistance and drug responsiveness, enabling a deeper understanding of these states. Our findings highlight the capacity of this approach to unveil genes that may not be identified as crucial in traditional analyses, thus providing valuable insights into the intricate mechanisms of disease progression. By contextualizing these genes within this framework, their precise roles in driving disease progression can be more accurately elucidated. This methodology opens up avenues for further exploration and sheds light on the potential applications of this approach in the study of TMEs across various contexts. For instance, a future study could include IDH wild-type gliomas, which could provide valuable comparative insights into glioma progression across different molecular subtypes.

## 5 Conclusion

In conclusion, our study demonstrates the utility of scRNA-seq data and multilayer signaling networks to investigate glioma progression. By comparing the intercellular and intracellular signaling networks of grade III and grade IV glioma, we identified key genes and interactions associated with disease progression. Furthermore, we showcased the potential of these genes as biomarkers for patient survival prediction and highlighted their potential applications in the development of targeted therapies. Our findings offer valuable insights into the complex molecular mechanisms underlying glioma progression and provide a foundation for future research aimed at developing more effective therapeutic strategies.

Despite the promising results, our study has several limitations that should be addressed in future research. Firstly, our analysis relies on computational predictions and existing databases, which may not capture all biological complexities or novel interactions. Secondly, our focus on grade III and IV gliomas, while clinically relevant, does not provide insights into earlier stages of glioma development. Future research should incorporate data from grade I and II gliomas to provide a more comprehensive understanding of cancer progression. Lastly, while we identified potential biomarkers and therapeutic targets, their clinical utility requires extensive experimental validation and clinical trials before they can be translated into practice. Future studies should focus on further validating the identified genes and interactions using experimental models, in order to solidify their significance as potential therapeutic targets. Additionally, incorporating complementary omics data, such as proteomics and metabolomics, would provide a more comprehensive understanding of glioma progression and unveil additional targets for therapeutic intervention. Expanding our approach to investigate other cancer types holds the potential to uncover novel biomarkers and therapeutic targets in diverse malignancies.

## Data Availability

The original contributions presented in the study are included in the article/Supplementary Material, further inquiries can be directed to the corresponding author.

## Abbreviations

scRNA-seq, single-cell RNA sequencing; TF, transcription factor; WHO, world health organization; TME, tumor microenvironment; PEA, pathway enrichment analysis; QC, quality control; ERCC, external RNA controls consortium; PCA, principal component analysis; RTKs, receptor tyrosine kinases; GBM, glioblastoma multiform; EGFR, epidermal growth factor receptor; CV, cross validation; SVM, support vector machine; RF, random forest; KNN, k-nearest neighbor; LR, linear regression; *PDGFRA*, platelet-derived growth factor receptor A; *PDGFA*, Platelet-derived growth factor A; *CREB1*, Cyclic AMP-responsive element-binding protein 1; *PLAT*, Plasminogen activator, tissue-type; tPA, tissue plasminogen activator; UMAP, uniform manifold approximation and projection; SMOTE, synthetic minority oversampling technique; CGGA, Chinese glioma genome atlas; TCGA, the cancer genome atlas.
