# Supplementary material for: Integrative analysis of single-cell transcriptomic and multilayer signaling networks in glioma reveal tumor progression stage
Source: Front Genet. 2024 Nov 13;15:1446903. doi: 10.3389/fgene.2024.1446903 (PMC11599185; doi:10.3389/fgene.2024.1446903)
Supplement: Supplementary file 3 [file Table1.DOCX]

Supplementary Material

# Supplementary Figures


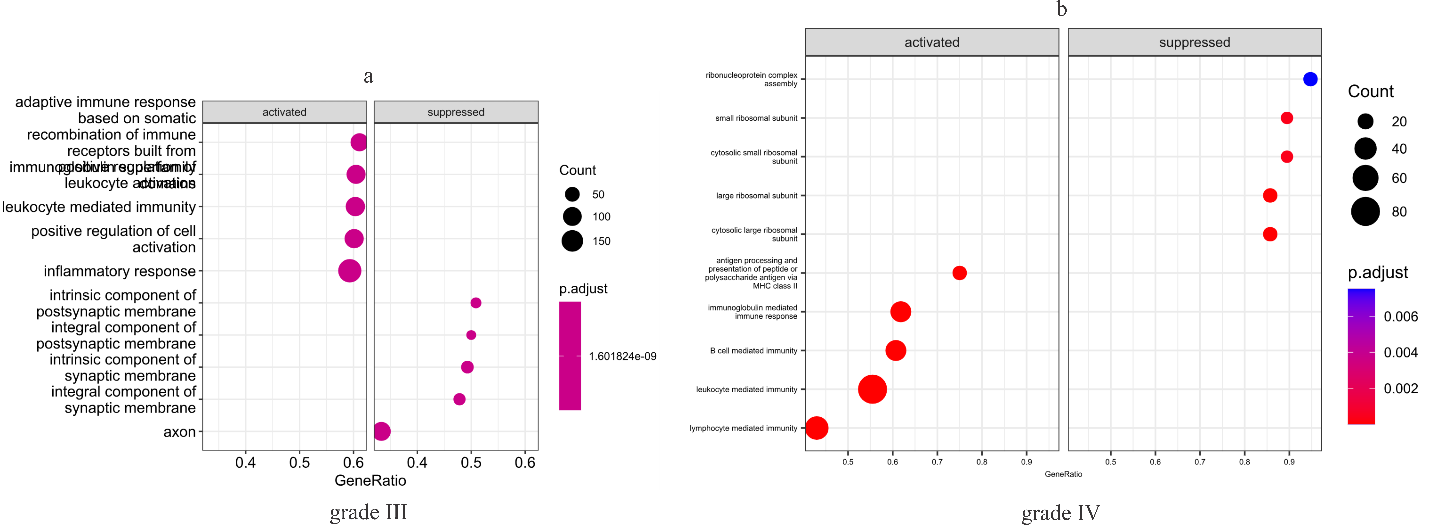


**Supplementary Figure 1.** Functional enrichment analysis of the marker genes in Cluster 1 for (a) grade III and (b) grade IV


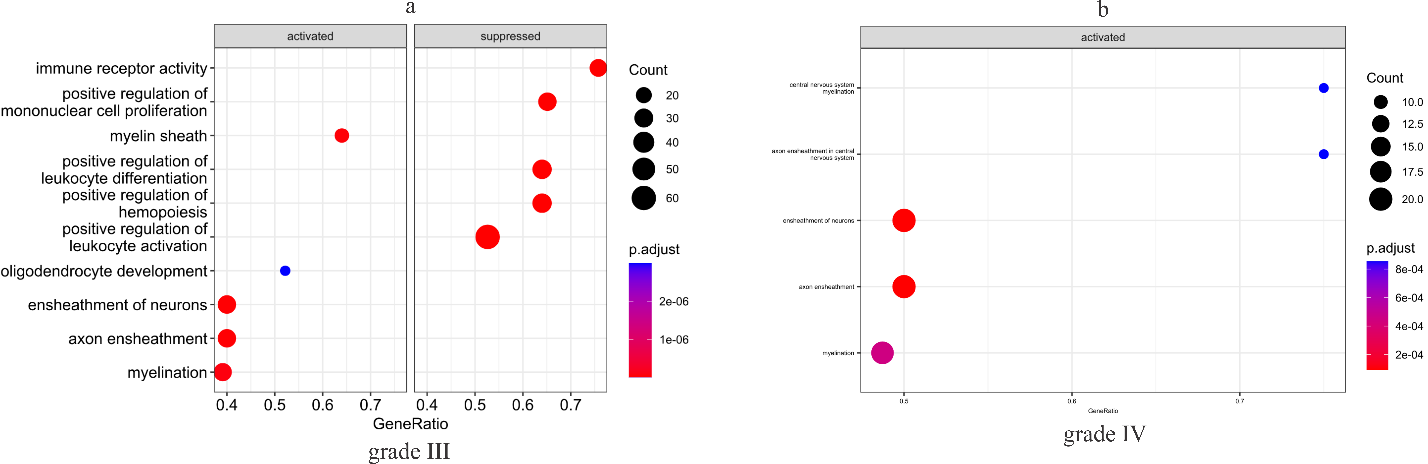


**Supplementary Figure 2.** Functional enrichment analysis of the marker genes in Cluster 2 for (a) grade III and (b) grade IV.


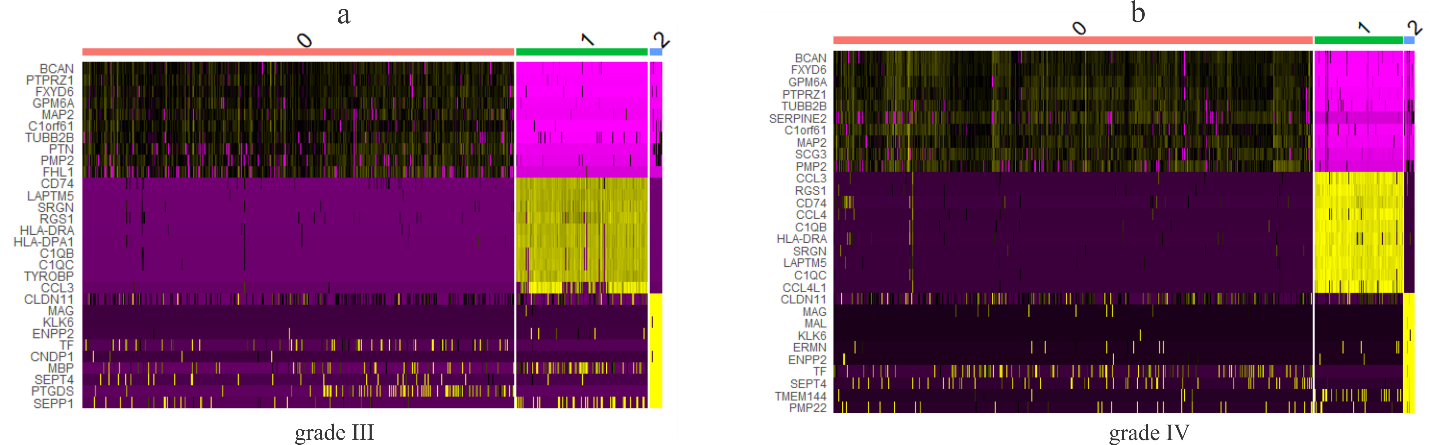


**Supplementary Figure 3.** The heatmap representation of each most significant marker for each cluster as additional support for the cell type annotation part for (a) grade III and (b) grade IV.
